# Relationship between Audiometric Slope and Tinnitus Pitch in Tinnitus Patients: Insights into the Mechanisms of Tinnitus Generation

**DOI:** 10.1371/journal.pone.0034878

**Published:** 2012-04-18

**Authors:** Martin Schecklmann, Veronika Vielsmeier, Thomas Steffens, Michael Landgrebe, Berthold Langguth, Tobias Kleinjung

**Affiliations:** 1 Department of Psychiatry and Psychotherapy, University of Regensburg, Regensburg, Germany; 2 Interdisciplinary Tinnitus Clinic, University of Regensburg, Regensburg, Germany; 3 Department of Otorhinolaryngology, University of Regensburg, Regensburg, Germany; 4 Department of Otorhinolaryngology, University of Zurich, Zurich, Switzerland; Linkoping University, Sweden

## Abstract

**Background:**

Different mechanisms have been proposed to be involved in tinnitus generation, among them reduced lateral inhibition and homeostatic plasticity. On a perceptual level these different mechanisms should be reflected by the relationship between the individual audiometric slope and the perceived tinnitus pitch. Whereas some studies found the tinnitus pitch corresponding to the maximum hearing loss, others stressed the relevance of the edge frequency. This study investigates the relationship between tinnitus pitch and audiometric slope in a large sample.

**Methodology:**

This retrospective observational study analyzed 286 patients. The matched tinnitus pitch was compared to the frequency of maximum hearing loss and the edge of the audiogram (steepest hearing loss) by t-tests and correlation coefficients. These analyses were performed for the whole group and for sub-groups (uni- vs. bilateral (117 vs. 338 ears), pure-tone vs. narrow-band (340 vs. 115 ears), and low and high audiometric slope (114 vs. 113 ears)).

**Findings:**

For the right ear, tinnitus pitch was in the same range and correlated significantly with the frequency of maximum hearing loss, but differed from and did not correlate with the edge frequency. For the left ear, similar results were found but the correlation between tinnitus pitch and maximum hearing loss did not reach significance. Sub-group analyses (bi- and unilateral, tinnitus character, slope steepness) revealed identical results except for the sub-group with high audiometric slope which revealed a higher frequency of maximum hearing loss as compared to the tinnitus pitch.

**Conclusion:**

The study-results confirm a relationship between tinnitus pitch and maximum hearing loss but not to the edge frequency, suggesting that tinnitus is rather a fill-in-phenomenon resulting from homeostatic mechanisms, than the result of deficient lateral inhibition. Sub-group analyses suggest that audiometric steepness and the side of affected ear affect this relationship. Future studies should control for these potential confounding factors.

## Introduction

There is a lot of evidence that tinnitus, the phantom perception of sound, is a consequence of neuroplastic alterations in the central auditory pathways [1]. These alterations are assumed to result from a dysbalance of excitatory and inhibitory mechanisms on many levels of the auditory pathways as a consequence of disturbed sensory input due to hearing loss [2]. Hearing loss can have multiple reasons within the peripheral auditory system. However, damage to cochlear structures represents the main cause of hearing loss in tinnitus subjects [3]. Defects of the cochlea can develop as a consequence of ageing, exposure to loud noise, cochlear ischemia, viral infections or ototoxic drugs [2]. In many cases middle and high frequencies are predominantly affected which leads to audiograms of characteristic shape. Different theories exist which try to explain the relationship of different types of hearing impairment and the perceived tinnitus frequency, the so-called tinnitus pitch.

One theory proposes that tinnitus results from an edge effect caused by an imbalance of lateral inhibition at the boundary of the region of normal and impaired hearing, the “edge frequency” [4]. It is assumed that a discontinuity of input along the tonotopic axis leads to a dysbalance in lateral inhibition, which in turn results in an increased firing rate and increased synchrony of the cortical representation of the edge frequency [5], finally resulting in an expansion of this area towards the deprived cortical area [6]. This theory implies that the perceived tinnitus frequency should be related to the edge frequency.

Alternatively, it has been suggested that tinnitus is caused by homeostatic mechanisms, which aim to compensate reduced sensory input by reduction of inhibitory and/or increase of facilitatory mechanisms. This model predicts that changes in the processing of neuronal activity occur predominantly in the frequency range of reduced sensory input, which finally results in ongoing increased neuronal activity and/or synchrony in the respective central auditory pathways [7], [8]. According to this theory the frequency of tinnitus perception should correspond to the frequency of hearing loss.

The relation between the individual audiometric slope and the perceived tinnitus pitch has been subject to different studies which have demonstrated somewhat inconsistent results. While some authors found a clear relationship between the tinnitus pitch and the edge frequency [9], [10], others showed that the pitch corresponds to the area where hearing is impaired [11], [12], [13], [14] and is in some specific cases congruent to the frequency of maximum hearing loss [15]. A third group could not demonstrate any correlation of audiogram shape and tinnitus pitch [16]. It has been suggested that such a relation may only exist in certain tinnitus sub-groups [14], [16]. In particular perceptual characteristics of tinnitus have been proposed to be relevant [14]. Thus, the small sample sizes and the lack of sub-group analysis in most studies may provide an explanation for the conflicting results in the literature. Thus, the aim of the present study was to evaluate the relationship of hearing with tinnitus pitch in a large population of tinnitus patients and to evaluate the role of certain sub-groups. One hitherto largely neglected issue is the question whether it is of relevance if tinnitus is perceived uni- or bilaterally. Former studies only investigated patients with bilateral tinnitus [10], [14]. The only study focusing on this issue did not find a clear association [16]. In relation to a recent study we were especially interested if there is an association of audiometric edge in patients with narrow-band tinnitus [14]. As former studies are highly inconsistent we additionally focused on sub-groups classified by the slope of the audiogram. Since findings from earlier studies are relatively inconsistent we resigned to formulate specific hypotheses. This study was presented in part at the Annual Meeting of the American Academy of Otolaryngology-Head and Neck Surgery, San Francisco, CA, September 10–14 2011.

## Methods

### Objectives

In this study we investigated in a large sample of 286 patients with tinnitus the relationship between the perceived tinnitus pitch and parameters derived from the pure tone audiogram such as the lower edge frequency and the maximum hearing loss frequency. The number of participants enabled us to perform sub-group analyses for bilateral vs. unilateral tinnitus, pure-tone vs. noise-like tinnitus, and patients with low vs. high steepness at the audiometric slope.

### Ethics Statement

All participating subjects gave written informed consent for analysis of their data and inclusion of their data in the Tinnitus Research Initiative (TRI) database project. The TRI database project has been approved by the ethical committee of the University of Regensburg.

### Sample

We analyzed patient data of the TRI database. The TRI database is a collaborative project of specialized tinnitus clinics following the approach to pool data in one international database [17] according to a consensus for patient assessment and outcome measurement [18]. We included only patients from the Regensburg center (Germany) in this study to ensure homogeneity of the audiometric procedures.

**Figure 1:**
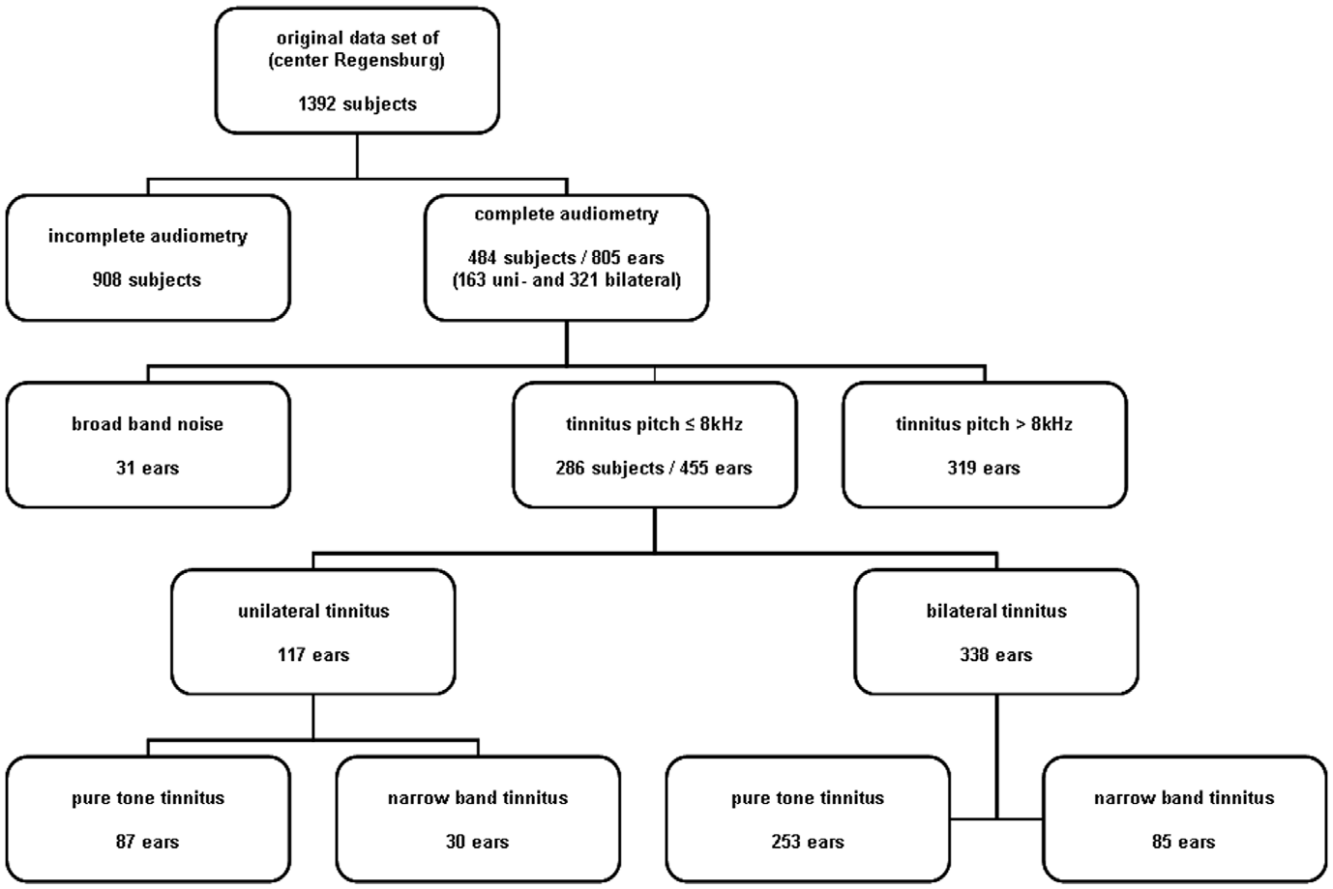
Study flow of inclusion of patients and ears.

The flow of data selection is displayed in figure 1. The automatic data export from the database on May^1st^ 2011 included 1392 subjects. First datasets from the Regensburg center with complete data of audiometry (0.125–8 kHz) and tinnitus matching at screening/baseline visits were selected reducing the sample to 484 subjects. In the following step patients with broad-band-noise-like tinnitus and with a tinnitus pitch above 8 kHz were excluded resulting in a data set of 286 patients and 455 ears respectively (117 subjects with uni- and 169 with bilateral tinnitus). The exclusion of patients with a tinnitus pitch above 8 kHz was motivated by the fact that audiometric data were only available up to 8 kHz. Distribution of patients with uni- and bilateral and with pure-tone and narrow-band tinnitus is shown in the bottom part of figure 1.

The 286 patients which were included in the analyses were 52.9±13.7 (17.1–86.5) years old; 92 (32.4%) subjects were female. Patients suffered for 90.7±93.9 (1–456.6) months from tinnitus. Ratings and questionnaires were 48.8±22.7 (4–94) for the German version of the Tinnitus Handicap Inventory [19], 42.5±17.4 (6–79) for the German version of the Tinnitus Questionnaire [20], and 6.3±2.1 (0–10) for tinnitus loudness in a numeric rating scale.

### Audiometry, pitch matching and calculation of audiogram parameters

Audiometry and tinnitus matching were done with a Madsen Itera (GN Otometrics, Germany) audiometer with Sennheiser HDA-200 supra-aural headphones (Sennheiser electronic GmbH & Co. KG, Germany). The hearing threshold for nine frequencies (0.125, 0.25, 0.5, 1, 2, 3, 4, 6, and 8 kHz) was determined by a standard Hughson-Westlake procedure (steps: 10 dB down, 5 dB up; 2 out of 3). In a second step, the frequency/pitch of the tinnitus was determined. In a first step, the pulsed tones of the audiogram where used to roughly match the tinnitus pitch and the patients were asked, whether the tinnitus sounds like a pure tone as just perceived during the audiometry, or does it sound like a broad band or a narrow band noise. To assist the comparison of the tinnitus pitch or sound quality either a pure tone or a white noise or a narrow band octave or 1/3-octave noise was presented to the ear with the tinnitus at nearly the same loudness as the tinnitus. If the pure tone threshold was too high to perceive a test signal at the side of the tinnitus, the contralateral better ear was used to present the sound. In case of a pure tone, a software sinus generator with a 1 Hz frequency resolution was then used in a bracketing procedure to match the pure tone pitch as exactly as possible. This technique is recommended for routine clinical use and seems to produce fewer octave confusions than others [21].

In case of a narrow band noise an octave or 1/3-octave filter bank with standard mid frequencies was used. The centre frequency was changed in the same way (1 Hz steps) as the bracketing procedure with pure tones until the narrow band noise matches the tinnitus pitch best. The center frequency of the matching signal was recorded as the pitch of the tinnitus.

The audiogram edge was defined as the lower frequency of two neighboring frequency pairs in the audiogram with the largest steepness, calculated as L(f2)−L(f1)/log2(f2/f1) [9]. If there were several frequency pairs with the same increase in hearing loss the lower frequency pair was used. To compute the mean audiogram edge across individuals, edge frequencies were first converted to a logarithmic scale, and then averaged, i.e. we used the geometric mean. The slope was indicated by the hearing level difference of this frequency pair (dB/octave).

In addition, the frequency of the maximum hearing loss was evaluated for each subject. If there were several frequencies with maximum hearing loss the lowest one was used. Averaging across individuals was done accordingly to the method used for the edge. All calculations were done with MatLab® (The Mathworks Inc., USA).

### Statistical analysis

The data analysis was based on data of the Tinnitus Research Initiative Database. Data management was conducted according to the Data Handling Plan (TRI-DHP V06, May 9^th^ 2011). Data analysis was conducted according to the Standard Operating Procedure (TRI-SA V01, May 9^th^ 2011), thereby following a study-specific Statistical Analysis Plan (SAP) that was written according to the SAP template (TRI-SAP-005, August 12^th^ 2011) published on http://database.tinnitusresearch.org.

As all statistical tests have to fulfill the assumption that all data points have to be independent observations we separated our analyses for the right and the left ear. By this approach one subject contributed only one data point or data from only one ear respectively for each statistical test. In a first step we contrasted the tinnitus pitch with the edge frequency and with the frequency of maximum hearing loss with Student t-tests for the whole group. Thereafter, we calculated Pearson correlation coefficients between tinnitus pitch and edge, and between tinnitus pitch and frequency of maximum hearing loss. In further steps we split the samples according to tinnitus laterality (bi- vs. unilateral), tinnitus character (pure-tone vs. narrow-band tinnitus), and steepness of the slope at the audiometric edge (according to two halves of the sample as obtained by median split, i.e., low vs. high slope) and repeated the analyses in these sub-groups. Thereafter, we contrasted the sub-groups (bi- vs. unilateral, pure-tone vs. narrow-band, lower vs. higher half of the sample) for tinnitus pitch, and frequency of maximum hearing loss. As analyses were performed in logarithmic scale, we just provide back-transformed mean data of frequency space in text and abstained from presenting standard deviations.

## Results

**Figure 2:**
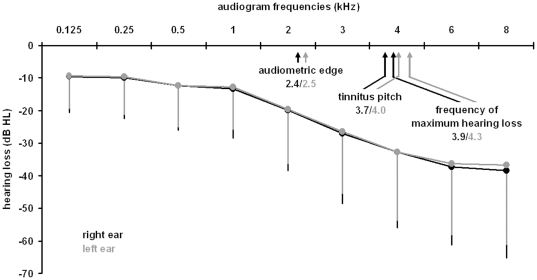
Relationship of audiogram and tinnitus pitch averaged for the whole sample, for the right and left ear. Please note that the averaged frequency of maximum hearing loss and the hearing loss as indicated by averaged audiogram data do not necessary result in the same values.

Numeric values for the frequencies/pitches for the whole group and sub-group analyses are given in table 1; statistical values for all calculations are given in table 2. Figure 2 depicts the frequency localization of the edge, the frequency of maximum hearing loss, the tinnitus pitch and the audiogram averaged for the whole group.

**Table 1:** Mean frequencies/pitches for the whole group and sub-groups (kHz).

	whole group		bilateral tinnitus		pure-tone tinnitus		low slope	
	right ear	left ear	right ear	left ear	right ear	left ear	right ear	left ear
tinnitus pitch	3.7	4.0	3.9	3.9	3.9	4.3	3.1	3.6
frequency of maximum hearing loss	3.9	4.3	4.1	4.2	3.9	4.3	2.5	3.3
audiometric edge	2.4	2.5	2.5	2.4	2.4	2.6	1.9	2.2

			unilateral tinnitus		narrow-band tinnitus		high slope	
			right ear	left ear	right ear	left ear	right ear	left ear
tinnitus pitch			3.2	4.3	3.4	3.2	4.6	4.5
frequency of maximum hearing loss			3.4	4.7	4.0	4.2	5.6	5.6
audiometric edge			2.1	2.8	2.3	2.3	2.9	2.8

**Table 2:** Contrasts and correlations of tinnitus pitch and frequency of maximum hearing loss for the whole group and sub-groups (significant or near-significant effects in bold font; T = student T; r = Pearson r).

	whole group				bilateral tinnitus				pure-tone tinnitus				low slope			
	right ear		left ear		right ear		left ear		right ear		left ear		right ear		left ear	
frequency	T (df = 211)	r (n = 212)	T (df = 242)	r (n = 243)	T (df = 166)	r (n = 167)	T (df = 170)	r (n = 171)	T (df = 158)	r (n = 159)	T (df = 180)	r (n = 181)	T (df = 105)	r (n = 106)	T (df = 120)	r (n = 121)
pitch - edge	**T = 5.324 p<0.001**	r = 0.043 p = 0.532	**T = 6.387 p<0.001**	r = −0.014 p = 0.827	**T = 4.992 p<0.001**	r = 0.029 p = 0.712	**T = 5.397 p<0.001**	r = 0.030 p = 0.692	**T = 4.881 p<0.001**	r = 0.005 p = 0.952	**T = 6.849 p<0.001**	r = 0.020 p = 0.789	**T = 2.966 p = 0.004**	r = −0.034 p = 0.732	**T = 3.943 p<0.001**	r = −0.067 p = 0.465
pitch – max. hearing loss	T = 0.489 p = 0.625	**r = 0.324 p<0.001**	T = 0.819 p = 0.413	r = 0.068 p = 0.201	T = 0.396 p = 0.693	**r = 0.236 p = 0.002**	T = 0.570 p = 0.569	r = 0.078 p = 0.312	T = 0.019 p = 0.985	**r = 0.267 p = 0.001**	T = 0.042 p = 0.966	r = 0.018 p = 0.892	T = 0.772 p = 0.442	**r = 0.240 p = 0.013**	T = 0.631 p = 0.530	r = 0.012 p = 0.897

					unilateral tinnitus				narrow-band tinnitus				high slope			
					right ear		left ear		right ear		right ear		right ear		left ear	
					T (df = 44)	r (n = 45)	T (df = 71)	r (n = 72)	T (df = 52)	r (n = 53)	T (df = 61)	r (n = 62)	T (df = 105)	r (n = 106)	T (df = 121)	r (n = 122)
pitch - edge					**T = 2.029 p = 0.049**	r = 0.052 p = 0.735	**T = 3.392 p = 0.001**	r = −0.185 p = 0.120	**T = 2.195 p = 0.033**	r = 0.128 p = 0.361	**T = 1.752 p = 0.085**	r = −0.090 p = 0.488	**T = 5.814 p<0.001**	r = 0.068 p = 0.489	**T = 5.621 p<0.001**	r = 0.026 p = 0.772
pitch - max. hearing loss					T = 0.303 p = 0.764	**r = 0.541 p<0.001**	T = 0.650 p = 0.518	r = 0.025 p = 0.834	T = 1.118 p = 0.269	**r = 0.487 p<0.001**	T = 1.605 p = 0.114	r = 0.188 p = 0.144	**T = 3.821 p = 0.001**	**r = 0.480 p<0.001**	**T = 2.751 p = 0.007**	r = 0.106 p = 0.246

					bi- vs. unilateral tinnitus		pure-tone vs. narrow-band tinnitus		lower vs. higher slope	
					right ear	left ear	right ear	left ear	right ear	left ear
					T (df = 210)	T (df = 241)	T (df = 210)	T (df = 241)	T (df = 210)	T (df = 241)
Pitch					T = 1.180 p = 0.239	T = 0.684 p = 0.495	T = 0.882 p = 0.379	**T = 2.160 p = 0.032**	**T = 2.914 p = 0.004**	**T = 1.918 p = 0.056**
Edge					T = 1.072 p = 0.285	T = 0.997 p = 0.320	T = 0.156 p = 0.876	T = 0.217 p = 0.829	**T = 5.307 p<0.001**	**T = 5.022 p<0.001**

In the whole group analysis tinnitus pitch had a significantly higher frequency than the audiometric edge, but did not differ significantly from the frequency of maximum hearing loss for both ears. In a second analyzes of the sample with narrow band noise like tinnitus we replaced the center frequency of the narrow band noise with the lower bound frequency which is closer to the audiometric edge. This did not cause any changes of the result as there was again only significant correlation of the tinnitus pitch with the maximum hearing loss frequency but not with the edge frequency. Tinnitus pitch correlated positively with the frequency of maximum hearing loss, but not with the audiogram edge for the right ear. For the left ear no significant associations of pitch with edge and frequency of maximum hearing loss was found.

Sub-group analyses for tinnitus laterality (bi- vs. unilateral) and tinnitus character (pure-tone vs. narrow-band) showed results similar to the whole group analyses, i.e., tinnitus pitch had a significant higher frequency than the edge for both ears, frequency of maximum hearing loss did not differ from the pitch frequency for both ears, and frequency of maximum hearing loss was positively correlated with the tinnitus pitch for the right ear. Contrasts between unilateral and bilateral tinnitus did not reveal a significant difference for tinnitus pitch and frequency of maximum hearing loss. Also pure-tone and narrow-band tinnitus did not differ in these variables except for tinnitus pitch with a near-threshold p-value.

Dividing the sample in two halves by median split according to the slope at the audiometric edge showed that tinnitus pitch and frequency of maximum hearing loss was higher for the high slope in contrast to the low slope. Again the findings were comparable to the whole group analyses. In addition, frequency of maximum hearing loss was significantly higher than tinnitus pitch for the high slope group.

## Discussion

We did not find an association between tinnitus pitch and audiometric edge in a sample of 286 patients (455 ears), but between pitch and frequency of maximum hearing loss, as indicated by t-tests and correlations. Frequency of maximum hearing loss and tinnitus pitch was in the same frequency range and tinnitus pitch had a higher frequency than the audiometric edge. In addition, there was a significant positive correlation between tinnitus pitch and frequency of maximum hearing loss for the right ear. For the left ear, pitch and frequency of maximum hearing loss was not correlated, although both were in the same frequency range. Edge and pitch frequency were not correlated. These findings were replicated in all analyzed sub-groups, i.e., bi- and unilateral tinnitus, pure-tone vs. narrow-band tinnitus, and tinnitus with low slope at the audiometric edge. The group with a high slope showed also a correlation between pitch and frequency of maximum hearing loss for the right ear, but in addition the frequency of maximum hearing loss was higher than tinnitus pitch which was higher than the edge frequency. Thus, our results do not support a relationship between edge frequency and tinnitus pitch, but rather confirm previous studies demonstrating that the tinnitus corresponds to the frequency region of hearing loss [11], [12], [13].

With respect to mechanisms involved in tinnitus generation our data favours homeostatic plasticity as the decisive mechanism of tinnitus generation [7], [8]. In detail, it has been proposed that the central auditory system maintains neural homeostasis to preserve stable mean firing and neural coding efficiency. In many cases of sensory deprivation, “neural noise” might be amplified due to the overall increase of gain and result in increased neuronal activity in the deprived frequency spectrum, which is finally perceived as tinnitus [7].

Hitherto, antecedent literature indicated ambiguous results regarding the relationship of audiometric edge and tinnitus pitch (for an overview until the year 2000 see Tyler et al. [22]). A relatively large recent study (n = 195) could not demonstrate a significant relationship between tinnitus pitch and the low frequency edge [16]. Similar to our study, tinnitus pitch (5.0 kHz) and frequency of maximum hearing loss (5.0 kHz) were in the same frequency range in contrast to the edge (2.2 kHz) which was lower. However, in contrast to our data tinnitus pitch and the frequency of maximum hearing loss were also not correlated. In several additional analyses, they tried, but failed to find associations of edge and pitch in sub-groups based on the slope of the audiogram, tinnitus character, or tinnitus laterality. This relationship was only evident in some single cases.

In another recent study, Sereda et al. [14] studied 67 subjects with bilateral tinnitus. The complete sample failed to demonstrate an association of tinnitus pitch and edge frequency, however the tinnitus pitch was located rather within the area of hearing loss than in the area of the edge frequency in all patients. In the sub-group of subjects with narrow-band noise like tinnitus (n = 23) a significant positive relationship was noted for the edge frequency and tinnitus pitch, with the pitch being more than an octave above the edge frequency. In contrast to our study the audiogram involved the high frequency range up to 16 kHz and the whole perceived tinnitus frequency composition was determined. It should also be noted, that narrow-band tinnitus in this study was defined as a frequency band of 0.13–0.25 kHz bandwidth which is different from our definition of narrow-band tinnitus (<1 octave). An association of tinnitus pitch and edge within sub-groups of different slopes (shallow, moderate, and steep) could not be determined.

König and colleagues [9] also reported that tinnitus pitch was on average 1.5±0.1 octaves above the edge frequency in a sub-group of 24 patients with tonal tinnitus out of 71 patients with bilateral noise induced hearing loss. This sub-group also showed a significant correlation of pitch and edge and of pitch and frequency of the steepest slope. In contrast to our study, slope and edge were independently defined and were not necessarily related to the same frequency pair.

A recent study of Moore et al. [10] revealed a definite association between the values of the tinnitus pitch and the edge frequency with a correlation of 0.94 in 11 subjects with bilateral tonal tinnitus. This was the first study, which could show that tinnitus pitch (1.5 kHz) and audiometric edge (1.5 kHz) were in the same range. The authors remark that results depend critically on training procedures in order to avoid an octave error in tinnitus matching. Thus, patients tend to rate the frequency of their tinnitus one octave too high. In general, tinnitus pitches were one or two octaves lower after training.

The findings of König et al. [9] and Moore et al. [10] are in contrast to our results of no relationship between tinnitus pitch and audiometric edge. We rather found an association with tinnitus pitch and frequency of maximum hearing loss. Even splitting the sample in sub-groups which are considered to be specific for edge-pitch associations could not reveal such an association. Neither uni- or bilateral, nor pure-tone or narrow-band tinnitus, nor low or high sloping audiograms indicated an association of pitch and edge. This is in contrast to the findings of Sereda et al. [14] who find an association of pitch and edge in the sub-group of patients with narrow-band tinnitus. Notably the definition of narrow-band tinnitus in that study was not identical to the definition used in our study.

In our study we did not use a particular tinnitus matching training as Moore and colleagues did. Thus, we cannot exclude that some of our subjects may have an octave error at tinnitus pitch matching. However, an octave error in some patients would produce a systematic effect and could eventually explain the observed difference between the edge frequency and the tinnitus pitch, but not the lack of correlation between these two factors.

One may also argue that in the majority of patients the discontinuity in the audiogram was not very high and may not be reflected by discontinuities at the inner hair cell level which might be the necessary precondition for an edge frequency effect. However, if the edge frequency effect only explains the generation of tinnitus in patients with an extremely steep audiometric slope, then it would not provide an explanation for tinnitus generation in the vast majority of tinnitus patients with moderate hearing loss. Moreover, we did not find any differences in the relationship between edge and tinnitus pitch between the low and high slope groups. Thus, our data gives no hint that the edge frequency effect may play a major role in tinnitus generation for a relevant sub-group of tinnitus patients.

It should be noted that we excluded patients with a tinnitus pitch above 8 kHz and with broad-band tinnitus, narrowing the external validity to a certain subpopulation (see also figure 2). The prevalence of broad-band tinnitus in our sample is low maybe due to lower distress in these patients. The prevalence of high-frequency tinnitus was rather high, thus, future studies should also focus on high-frequency audiogram data. It should also be considered that definition of pure-tone or narrow-band tinnitus was done according to an audiological pitch matching procedure as there is incongruence between verbal descriptions and matching procedures. We are well aware of the fact that all pitch matching procedures have some limitations, e.g. octave error, intra-individual variation and reproducibility. An international consensus on standardized test procedures is still lacking [23]. However, with the bracketing method we chose a feasible technique which is recommended for routine clinical use in tinnitus patients [21].

In addition we found a lateralization effect. Whereas for the right ear pitch and frequency of maximum hearing loss were in the same frequency range and were correlated, for the left ear pitch and frequency of maximum hearing loss were in the same frequency range, but were not significantly correlated. Lying in the same range in a correlated manner provides a strong hint that there is a close relationship between both measures. This fits very well to the central gain hypothesis (see above). Lying in the same frequency range without correlating depicts only a coarse relationship suggesting that the neurophysiological basis of tinnitus may depend on its laterality. Further support for a lateralization effect comes from epidemiological studies showing a higher proportion of left-sided unilateral tinnitus especially in tinnitus patients with hearing loss [24] and from electrophysiological studies showing differences in laterality indices for the right and left ear [25]. However, exact mechanisms are speculative and our findings should be considered preliminary till confirmed by further studies. Nevertheless tinnitus laterality should be considered as a potential confounding factor in future studies. It is also of relevance to consider this issue methodologically, as using both ears in one analysis might introduce statistical errors as the assumption of independence of data points is not fulfilled. This was not done for most studies in this context.

Furthermore, for the high slope tinnitus group frequency of maximum hearing loss was higher than tinnitus pitch although both measures were correlated. This is in line with animal models of noise induced tinnitus which show a tinnitus pitch above the frequency of the presented noise [26]. This again might indicate another neurophysiological mechanism.

In summary, our findings are in line with most previous publications by indicating a relationship between tinnitus pitch and audiogram variables. The observed association of tinnitus pitch with the frequency of maximum hearing loss provides further support for the hypothesis of homeostatic plasticity as the relevant mechanism for tinnitus generation [7], [8]. However, this model might not account completely for all subjects, especially for left-sided tinnitus and tinnitus patients with steep audiograms. This information is also relevant for illustrating the relationship of hearing loss and tinnitus in patient education and may guide the recommendation for the use of hearing aids for treating tinnitus accompanied by hearing loss [27]. We are aware that the present findings just represent a correlational, and not a causal relationship of tinnitus pitch and frequency of maximum hearing loss. Therefore the data should be complemented by further large interventional studies investigating the effect of hearing aids on the frequency composition of the tinnitus percept [28].

## References

[1] De Ridder D, Elgoyhen AB, Romo R, Langguth B (2011). Phantom percepts: tinnitus and pain as persisting aversive memory networks.. Proc Natl Acad Sci U S A.

[2] Møller AR, Sismanis A (2003). Pathophysiology of Tinnitus.. Otolaryngol Clin N Am.

[3] Roberts LE, Eggermont JJ, Caspary DM, Shore SE, Melcher JR (2010). Ringing ears: the neuroscience of tinnitus.. J Neurosci.

[4] Kiang NY-S, Moxon EC, Levine PA, Wolstenholme GEW, Knight J (1970). Auditory-nerve activity in cats with normal and abnormal cochleas.. Sensorineural hearing loss.

[5] Llinas RR, Ribary U, Jeanmonod D, Kronberg E, Mitra PP (1999). Thalamocortical dysrhythmia: A neurological and neuropsychiatric syndrome characterized by magnetoencephalography.. Proc Natl Acad Sci.

[6] Robertson D, Irvine DR (1989). Plasticity of frequency organization in auditory cortex of guinea pigs with partial unilateral deafness.. J Comp Neurol.

[7] Norena AJ (2011). An integrative model of tinnitus based on a central gain controlling neural sensitivity.. Neurosci Biobehav Rev.

[8] Schaette R, Kempter R (2009). Predicting tinnitus pitch from patients' audiograms with a computational model for the development of neuronal hyperactivity.. J Neurophysiol.

[9] Konig O, Schaette R, Kempter R, Gross M (2006). Course of hearing loss and occurrence of tinnitus.. Hear Res.

[10] Moore BC, Vinay, Sandhya (2010). The relationship between tinnitus pitch and the edge frequency of the audiogram in individuals with hearing impairment and tonal tinnitus.. Hear Res.

[11] Henry JA, Meikle MB (1999). Pulsed versus continuous tones for evaluating the loudness of tinnitus.. J Am Acad Audiol.

[12] Norena A, Micheyl C, Chery-Croze S, Collet L (2002). Psychoacoustic characterization of the tinnitus spectrum: implications for the underlying mechanisms of tinnitus.. Audiol Neurootol.

[13] Roberts LE, Moffat G, Bosnyak DJ (2006). Residual inhibition functions in relation to tinnitus spectra and auditory threshold shift.. Acta Otolaryngol Suppl.

[14] Sereda M, Hall DA, Bosnyak DJ, Edmondson-Jones M, Roberts LE (2011). Re-examining the relationship between audiometric profile and tinnitus pitch.. Int J Audiol.

[15] Ochi K, Ohashi T, Kenmochi M (2003). Hearing impairment and tinnitus pitch in patients with unilateral tinnitus: comparison of sudden hearing loss and chronic tinnitus.. Laryngoscope.

[16] Pan T, Tyler RS, Ji H, Coelho C, Gehringer AK (2009). The relationship between tinnitus pitch and the audiogram.. Int J Audiol.

[17] Landgrebe M, Zeman F, Koller M, Eberl Y, Mohr M (2010). The Tinnitus Research Initiative (TRI) database: a new approach for delineation of tinnitus subtypes and generation of predictors for treatment outcome.. BMC Med Inform Decis Mak.

[18] Langguth B, Goodey R, Azevedo A, Bjorne A, Cacace A (2007). Consensus for tinnitus patient assessment and treatment outcome measurement: Tinnitus Research Initiative meeting, Regensburg, July 2006.. Prog Brain Res.

[19] Kleinjung T, Fischer B, Langguth B, Sand PG, Hajak G (2007). Validation of the German-version Tinnitus Handicap Inventory (THI).. Psychiatrische Praxis.

[20] Goebel G, Hiller W (1994). [The tinnitus questionnaire. A standard instrument for grading the degree of tinnitus. Results of a multicenter study with the tinnitus questionnaire].. HNO.

[21] Tyler RS, Conrad-Armes D (1983). Tinnitus pitch: a comparison of three measurement methods.. Br J Audiol.

[22] Tyler RS, Tyler RS (2000). Psychoacoustical measurement.. Tinnitus Handbook.

[23] Henry JA, Rheinsburg B, Owens KK, Ellingson RM (2006). New instrumentation for automated tinnitus psychoacoustic assessment.. Acta Otolaryngol Suppl.

[24] Martines F, Bentivegna D, Martines E, Sciacca V, Martinciglio G (2010). Characteristics of tinnitus with or without hearing loss: clinical observations in Sicilian tinnitus patients.. Auris Nasus Larynx.

[25] Hine DW, Honan CA, Marks AD, Brettschneider K (2007). Development and validation of the Smoking Expectancy Scale for Adolescents.. Psychol Assess.

[26] Kaltenbach JA (2011). Tinnitus: Models and mechanisms.. Hear Res.

[27] Schaette R, Konig O, Hornig D, Gross M, Kempter R (2010). Acoustic stimulation treatments against tinnitus could be most effective when tinnitus pitch is within the stimulated frequency range.. Hear Res.

[28] Moffat G, Adjout K, Gallego S, Thai-Van H, Collet L (2009). Effects of hearing aid fitting on the perceptual characteristics of tinnitus.. Hear Res.

